# Changes in seasonality and sex ratio of scrub typhus: a case study of South Korea from 2003 to 2019 based on wavelet transform analysis

**DOI:** 10.1186/s12879-024-09858-0

**Published:** 2024-09-28

**Authors:** Jeehyun Kim, Penelope Vounatsou, Byung Chul Chun

**Affiliations:** 1 Department of Preventive Medicine, Korea University College of Medicine, Seoul, Republic of Korea; 2 Transdisciplinary Major in Learning Health Systems, Department of Healthcare Sciences, Graduate School, Korea University, Seoul, Republic of Korea; 3 Swiss Tropical and Public Health Institute, University of Basel, Basel, Switzerland

**Keywords:** Scrub typhus, *Orientia tsutsugamushi*, Wavelet analysis, Seasons, Sex ratio, Vector-Borne diseases, Mites, Climate change

## Abstract

**Background:**

Scrub typhus (ST, also known as tsutsugamushi disease) is a common febrile vector-borne disease in South Korea and commonly known as autumn- and female-dominant disease. Although understanding changes in seasonality and sex differences in ST is essential for preparing health interventions, previous studies have not dealt with variations in periodicity and demographic characteristics in detail. Therefore, we aimed to quantify the temporal dynamics of seasonal patterns and sex differences in the incidence of ST in South Korea.

**Methods:**

We extracted epidemiological week (epi-week)-based ST cases from 2003 to 2019 Korean National Health Insurance Service data (ICD-10-CM code: A75.3). To determine changes in seasonality and sex differences, year-, sex-, and age-group-stratified male-to-female ratios and wavelet transform analyses were conducted.

**Results:**

Between 2003 and 2019, 213,976 ST cases were identified. The incidence per 100,000 population increased by 408.8% from 9.1 in 2003 to 37.2 in 2012, and subsequently decreased by 59.7% from 2012 to 15.0 in 2019. According to the continuous wavelet transform results, ST exhibited a dual seasonal pattern with dominant seasonality in autumn and smaller seasonality in spring from 2005 to 2019. Overall, the periodicity of seasonality decreased, whereas its strength decreased in autumn and increased in spring. With an overall male-to-female ratio being 0.68:1, the ratio has increased from 0.67:1 in 2003 to 0.78:1 in 2019 (Kendall’s τ = 0.706, p < 0.001). However, interestingly, the ratio varied significantly across different age groups.

**Conclusions:**

Our findings quantitatively demonstrated changes in seasonality with dual seasonal pattern and shortened overall periodicity and a decrease in sex differences of ST in South Korea. Our study suggests the need for continuous surveillance on populations of vector and host to address ST dynamics to preemptively prepare against global warming.

**Supplementary Information:**

The online version contains supplementary material available at 10.1186/s12879-024-09858-0.

## Background

Scrub typhus (ST), also known as tsutsugamushi disease, is a febrile vector-borne disease caused by an intracellular bacterium, *Orienta tsutsugamushi* [1]. The chigger, the larval stage of the trombiculid mite, is a known vector and reservoir of the disease, and rodents are its natural hosts [2]. Currently, no vaccine for ST is available and reinfection is possible [1]. The clinical manifestations of ST, which encompass a range of nonspecific influenza-like symptoms, including fever, headache, and eschar [1], can progress to severe complications that threaten life if left untreated or not addressed promptly [1, 2]. Among the countries in the tsutsugamushi triangle—a geographical cluster of ST incidence in the Asia-Pacific region spanning northern Japan to Pakistan and Afghanistan in the west and to northern Australia in the south [1], South Korea exhibits distinct characteristics of ST in periodicity and demographic factors.

Among the distinct features of ST in South Korea, the extent of change in seasonality should be thoroughly investigated. Some studies have suggested potential seasonal patterns of ST in spring [3], while it is recognized as a typical rodent-borne disease of the autumn in South Korea [4–6], similar to the pattern observed in Japan [1]. For vector-borne diseases, tracking the dynamics of seasonality using long-term stable data is essential to provide key information for early warning systems, which is crucial for determining the appropriate timing for health interventions, especially in the era of global warming [7].

Moreover, while the typical global trend of ST shows a neutral sex ratio [1], the situation in the South Korea is characterized by a female-dominant pattern [1, 3]. Specifically, according to the results of a meta-analysis, China and Japan have similar incidence rates between males and females, while Thailand has higher incidence in males [1]. As investigations into the relative prevalence influenced by host characteristics, such as demographic and regional factors, along with their temporal variations, are recommended to enhance the understanding of vector-borne diseases [8], South Korea-specific features of being female-dominant should be explored for a clear description of the disease and accurate analytic epidemiology [2]. Furthermore, exploring South Korea-specific features of being female-dominant would enrich our understanding of global ST patterns. Therefore, our study aimed to quantify the temporal dynamics in seasonality and sex differences of ST in South Korea using nationally representative long-term data.

## Methods

### Materials

We extracted ST cases from 2003 to 2019 from the Korean National Health Insurance Service (KNHIS) data, stratified by sex and epidemiologic week (epi-week). The KNHIS, South Korea’s single insurer under mandatory health insurance for all Korean citizens [9], provides data through healthcare utilization claims that are considered representative of the national population [9]. The eligibility information within the KNHIS data allows simultaneous stratification by epi-week and demographic features such as sex and age groups, crucial for our study aimed at observing temporal changes in ST, a condition predominantly affecting females in Korea. Insurance claim data primarily serve billing purposes rather than medical records, allowing for provisional diagnoses to facilitate clinical operations. In contrast, the National Infectious Disease Surveillance system relies on reported data for notifiable diseases, which may be subject to modifications in reporting criteria and variations in reporting rates [10]. Notably, in 2019, changes in reporting criteria for ST to exclude suspected cases from reporting requirements, resulting in a substantial 39.9% reduction in reported cases compared to the previous year [11], while the reduction in claim data was only 14.3%. As ST could be clinically diagnosed without confirmatory tests during outbreaks in endemic countries [12], although definitive diagnosis requires testing, changes in reporting criteria likely had a significant impact on reported cases but had limited effect on patient numbers and consequently on health insurance data. Therefore, to examine temporal trends of ST using stable long-term data with appropriate stratification, we employed KNHIS data. For sensitivity analysis, ST case counts were extracted from the notifiable disease surveillance reports available on the Infectious Disease Portal of the Korea Disease Control and Prevention Agency (https://dportal.kdca.go.kr), acknowledging that this data may have limitations due to reporting bias.

ST was defined according to the International Classification of Diseases, Tenth Revision, Clinical Modification code (ICD-10-CM code) A75.3. The data included information on residential address coded by municipality, sex (male and female), and age group, categorized in twenty-year intervals (0–19, 20–39, 40–59, 60–79, and ≥ 80 years of age). Patients with complete data were included in the analysis. We set ST incidence per 100,000 people using population data from Statistics Korea (https://kosis.kr). The incidence of the 53rd week of the years 2003, 2008, and 2014 was averaged over the 52nd week of the corresponding year for comparability across years. The Institutional Review Board (IRB) of Korea University granted an exemption for this study (IRB exemption number: KUIRB-2021-0237-02) as the data did not contain any personal identification information.

### Descriptive analysis

The case count and ST incidence per 100,000 population were determined by sex, age groups, and years by calendar year to provide basic information and explore sex and age differences in ST. The male-to-female ratio of ST incidence was described by year and age group. To statistically evaluate the temporal trend in the sex ratio of ST incidence, the Mann-Kendall test was utilized [13, 14]. The null hypothesis of the Mann-Kendall test posits that ST incidence is distributed independently, whereas the alternative hypothesis assumes a monotonic trend in ST incidence [13, 14].

Furthermore, a basic temporal additive decomposition of the log-transformed incidence from 2003 to 2019 was conducted to describe the trends, seasonality, and random variation. Initially, temporal additive decomposition was conducted by extracting the trend component through a moving average technique. Subsequently, the seasonal component was calculated by averaging over all periods and centering it [15]. The error component was then isolated by subtracting both the trend and seasonal components from the original data [15].

### Continuous wavelet transform analysis (CWT)

CWT on the log-transformed 2003–2019 ST incidence was conducted to detect seasonal trends. The CWT is a spectral temporal decomposition analysis that has the advantage of detecting seasonality in non-stationary data, which is a typical characteristic of epidemiological time-series data. Moreover, it has better localization in both time and frequency than does windowed Fourier decomposition, thereby enabling the detection of the transient nature of epidemiological data [16]. CWT also provides a more accurate representation of the underlying seasonality compared to traditional methods that may require pre-processing steps, such as detrending [16]. Additionally, CWT can capture patterns at different scales, which is essential in infectious disease epidemiology, where various seasonal drivers may operate on different time scales [16]. This enables the detection of multiple peaks within a year, which might not be visible through trend analysis alone.

The CWT was conducted using the Morlet wavelet, which is the default option of the WaveletComp package version 1.1 [17] in R (version 4.2.1; R Foundation, Vienna, Austria, https://cran.r-project.org), and 2,000 simulations to assess statistical significance with 95% confidence levels.

The null hypothesis posited that the observed time-series variability was indistinguishable from that expected from a purely random process [16]. The mother Morlet wavelet $\:\psi\:\left(t\right)$ was defined as follows, where $\:t$ denotes time and $\:\omega\:$ denotes angular frequency, which is set as 6 [17]:

$$\:\psi\:\left(t\right)={{\pi\:}^{-1/4}e}^{i\omega\:t}{e}^{-{t}^{2}/2}$$

The wavelet transform of a time-series $x(t)$ was defined as

$$\:{W}_{x}\left(\tau\:,s\right)=\sum\:_{t}x\left(t\right)\frac{1}{\sqrt{s}}\psi\:*\left(\frac{t-\tau\:}{s}\right)$$

where $\:\tau\:$ and s denote time shift and scale, respectively [17]. The use of the complex conjugate form ‘*’ enabled the interpretation of the wavelet transform as a cross-correlation of $x(t)$ with a set of wavelet daughters [16]. These daughters, derived from the mother wavelet, possess varying widths or scales $\:s$ at different positions $\:\tau\:$ [16].

The outcomes of the CWT were presented through a wavelet power spectrum plot and the average wavelet power of the time-series. The wavelet power spectrum plot depicts the degree of seasonality by time with two dimensions: calendar date on the x-axis and period of epi-week on the y-axis. If the time-series and the wavelet match well at the corresponding time and scale, the value of the wavelet power spectrum $$\:{S}_{x}$$$$\:(\tau\:,\:s)$$ becomes high [16, 17]. By contrast, if the matching of the time-series and wavelet is poor, the value of the wavelet power spectrum decreases [16, 17]. We presented the maxima of the wavelet power spectrum undulations with its 95% significance levels and regions with a boundary effect where information could be inaccurate [16, 17].

An average wavelet power determined whether an overall annual periodicity was present. Peaks were detected in the average wavelet power results. Subsequently, we extracted the maximum amount of power and the corresponding periodicity from each peak, based on a 95% significance level. Subgroup analysis using non-log-transformed data was conducted by sex and year groups. To identify shifts in CWT, the year group was divided at 2013 (comprising 2003–2012 and 2013–2019) because 2012 exhibited the highest incidence. Sensitivity analyses were performed using non-log-transformed epi-week data, data that maintained the information from the 53rd week of 2003, 2008, and 2014, and notifiable disease report data.

## Results

### Descriptive analysis

A total of 213,976 patients with ST without missing values among the 214,046 cases extracted from 2003 to 2019 were included in the study. Table 1 presents the case counts and ST incidence per 100,000 population by calendar year. Among all study participants, the incidence increased by approximately 408.8% from 9.1 in 2003 to 37.2 in 2012, and subsequently decreased by 59.7% from 2012 to 15.0 in 2019. Notably, the highest incidence was observed in 2012 among all participants with higher rates in both sexes (total sex, 37.2; male, 30.4; female, 43.9). Moreover, the ST case count in females was consistently higher than that in males between 2003 and 2019 (males, 86,484 [40.4%]; females, 127,492 [59.6%]). The 60–79 years of age group had the highest case count (104,628 [48.9%]), followed by the 40–59 (69,385 [32.4%]), ≥ 80 (17,573 [8.2%]), 20–39 (17,256 [8.1%], and 0–19 years of age groups (5,134 [2.4%], Table S1).

**Table 1:** Scrub typhus case count and incidence per 100,000 population by year

	Case count, *N* (%)				Incidence per 100,000 population			
Year	Total	Male	Female	Male-to-female ratio	Total	Male	Female	Male-to-female ratio
2003	4396	1776 (40.4)	2620 (59.6)	0.68	9.1	7.3	10.9	0.67
2004	11,034	4355 (39.5)	6679 (60.5)	0.65	22.8	17.9	27.7	0.65
2005	11,797	4457 (37.8)	7340 (62.2)	0.61	24.3	18.3	30.3	0.60
2006	10,237	3944 (38.5)	6293 (61.5)	0.63	20.9	16.1	25.8	0.62
2007	8113	3114 (38.4)	4999 (61.6)	0.62	16.5	12.6	20.3	0.62
2008	11,424	4420 (38.7)	7004 (61.3)	0.63	23.1	17.8	28.4	0.63
2009	15,502	6134 (39.6)	9368 (60.4)	0.65	31.2	24.6	37.7	0.65
2010	13,230	5365 (40.6)	7865 (59.4)	0.68	26.2	21.2	31.2	0.68
2011	13,700	5465 (39.9)	8235 (60.1)	0.66	27.0	21.5	32.5	0.66
2012	18,942	7766 (41.0)	11,176 (59.0)	0.69	37.2	30.4	43.9	0.69
2013	18,822	7742 (41.1)	11,080 (58.9)	0.70	36.8	30.3	43.4	0.70
2014	14,063	5705 (40.6)	8358 (59.4)	0.68	27.4	22.2	32.6	0.68
2015	15,719	6286 (40.0)	9433 (60.0)	0.67	30.5	24.4	36.6	0.67
2016	17,086	7191 (42.1)	9895 (57.9)	0.73	33.1	27.8	38.3	0.73
2017	13,262	5608 (42.3)	7654 (57.7)	0.73	25.6	21.7	29.5	0.73
2018	8879	3769 (42.4)	5110 (57.6)	0.74	17.1	14.6	19.7	0.74
2019	7770	3387 (43.6)	4383 (56.4)	0.77	15.0	13.1	16.9	0.78
Total	213,976	86,484 (40.4)	127,492 (59.6)	0.68	25.0	20.6	30.5	0.68

**Fig. 1:**
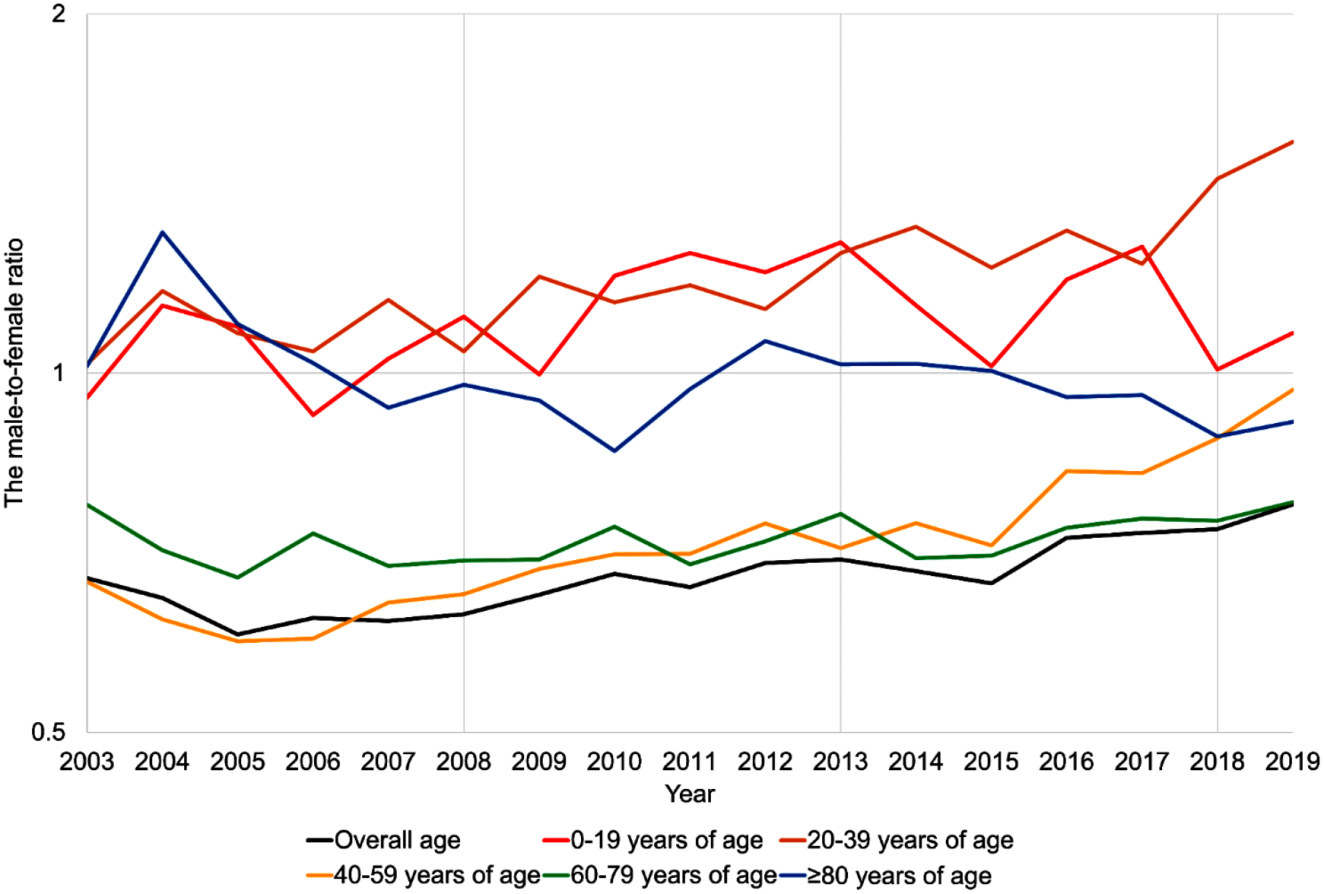
Male-to-female ratio of incidence per 100,000 population by age group. The black, red, orange, yellow, green, and blue lines refer to the male-to-female ratios for the overall study subjects, 0–19 years, 20–39 years, 40–59 years, 60–79 years, and ≥ 80 years of age, respectively

The male-to-female incidence ratio was 0.68:1 throughout the study period; however, the ratio increased between 2003 and 2019 (from 0.67:1 in 2003 to 0.78:1 in 2019; Kendall’s $\:\tau\:$=0.706, *p* < 0.001; Table 1). Furthermore, the sex ratio differed according to the age group (Fig. 1 and Table S2). Specifically, the 40–59 and 60–79 years of age groups were female-dominant, while the 0–19 and 20–39 years of age groups were male-dominant, and the ≥80 years-of-age group did not have sex differences. The sex ratios of 20–39, 40–59, and 60–79 years-of-age group increased, while the ratio of 0–19 and ≥80 years-of-age group did not increase.

**Fig. 2:**
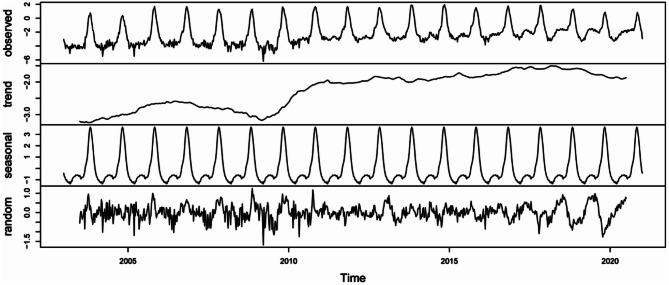
Results of additive decomposition of log-transformed scrub typhus incidence per 100,000 population in the epi-week

Figure 2 presents the observed time-series and trend, seasonality, and random variation of the log-transformed incidence. The log-transformed incidence showed an overall increase from 2003 to 2020 and increased approximately every five years. In both sexes, autumn exhibited significant seasonality, while spring showed comparatively lower seasonality (see Fig. S1).

### Continuous wavelet transform (CWT)

**Fig. 3:**
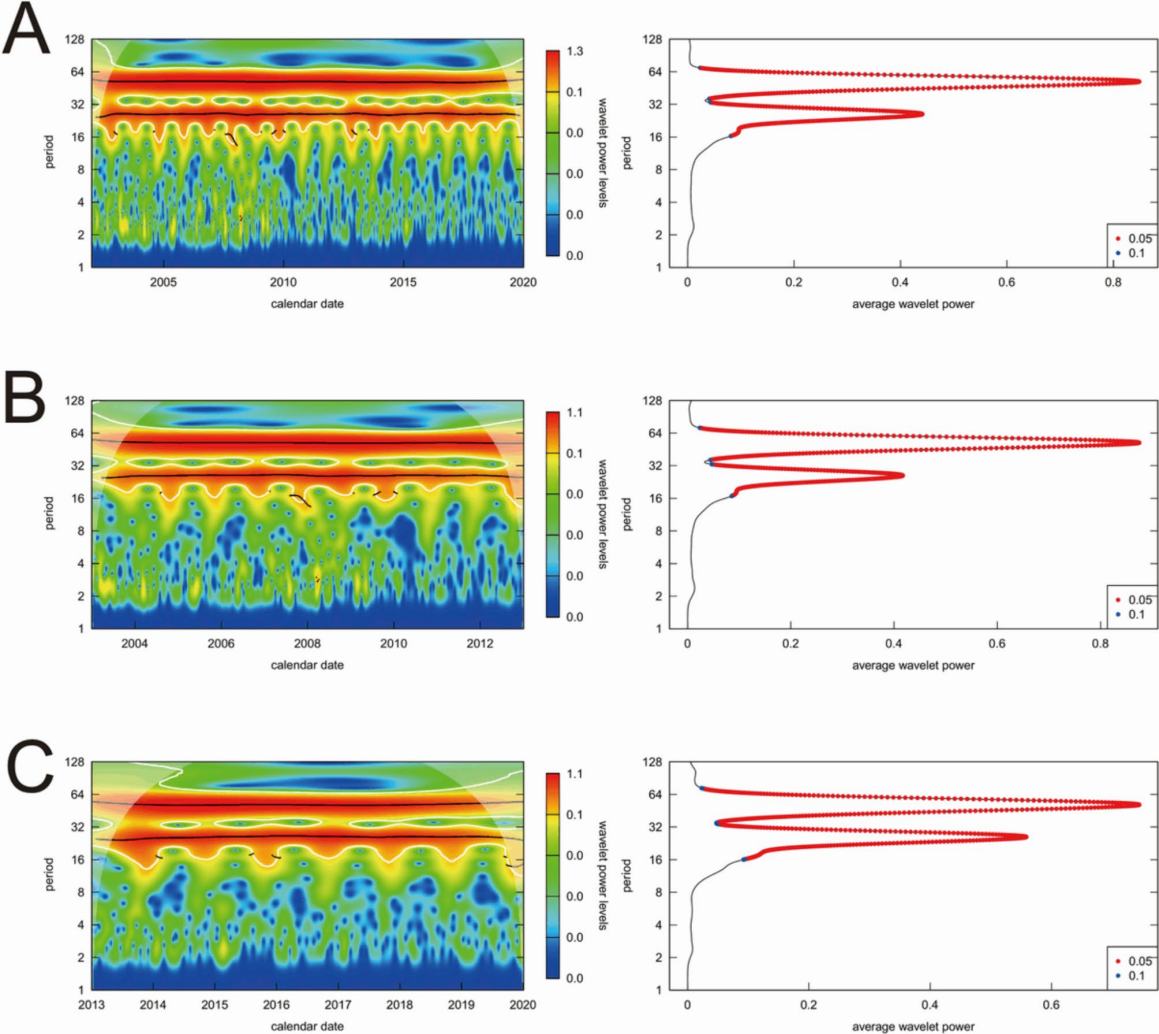
Results of the continuous wavelet transform on scrub typhus incidence in the epi-week by temporal stratification. The results are presented for **(A)** the total study subjects during the entire study period; **(B)** the total study subjects for the years 2003–2012; and **(C)** the total study subjects for the years 2013–2019. Left part of each panel: The color gradient from dark red to dark blue indicates wavelet power levels at corresponding times and periods. Dark red signifies high wavelet power levels, while dark blue indicates low levels, with darker red denoting a stronger degree of seasonality than darker blue. The black line represents the maxima of wavelet power spectrum undulations. White lines indicate the 95% significance levels, and the opaque area highlights the region with potential boundary effects, suggesting possible inaccuracies. Right part of each panel: The black line shows the average wavelet power for each period. Red dots mark the 95% significance levels, and blue dots represent the 90% confidence levels

The results of the CWT, based on the log-transformed incidence by year group, are presented in Fig. 3; Table 2. The wavelet power spectrum plot depicts two continuous seasonality periods of 52 and 26 weeks from 2005 to 2019, highlighted in dark red (left side of Fig. 3, Panel A). Furthermore, by examining the maximum power and corresponding periodicity at each peak, the dominant seasonality, attributed to the autumn season, was identified with a periodicity of 51.840 weeks, while the second-largest seasonality, associated with spring, showed a periodicity of 25.992 weeks (right side of Fig. 3, Panel A).

**Table 2:** The results of continuous wavelet transform on scrub typhus incidence per 100,000 population by sex and year group, expressed through the maximum amount of power (the corresponding periodicity)

	Total study period (2003–2019)	Period 1 (2003–2012)	Period 2 (2013–2019)
Log-transformed incidence			
The biggest seasonality	0.857 (51.840)	0.875 (52.128)	0.745 (51.411)
The second-biggest seasonality	0.448 (25.992)	0.417 (25.992)	0.559 (25.920)
Non-log-transformed male incidence			
The biggest seasonality	0.517 (51.984)	0.437 (51.984)	0.442 (51.697)
The second-biggest seasonality	0.415 (25.992)	0.365 (25.992)	0.396 (25.920)
Non-log-transformed female incidence			
The biggest seasonality	0.489 (51.984)	0.422 (51.984)	0.416 (51.840)
The second-biggest seasonality	0.412 (25.992)	0.368 (25.992)	0.395 (25.920)

The dominant seasonality among the total study subjects exhibited a decrease in power (from 0.875 in 2003–2012 to 0.745 in 2013–2019) and a reduction in length (from 52.128 weeks in 2003–2012 to 51.411 weeks in 2013–2019). Conversely, the second-largest seasonality increased in number (from 0.417 in 2003–2012 to 0.559 in 2013–2019), while also becoming shorter (25.992 weeks in 2003–2012, 25.920 weeks in 2013–2019, Fig. 3 Panel B and Panel C). The periodicity of seasonality decreased in both seasons, while the power of second-largest seasonality exhibited an increase.

However, the power of dominant seasonality in males increased (0.437 in 2003–2012, 0.442 in 2013–2019), unlike it decreased in females (0.422 in 2003–2012, 0.416 in 2013–2019; Table 2). Similarly, the results of the sensitivity analysis using non-log-transformed data (Fig. S2), data that maintained the information of the 53rd week (Fig. S3), and notifiable disease report data (Fig. S4) followed the trend of the main results (Fig. S2, Fig. S3, and Fig. S4).

## Discussion

We investigated the temporal and demographic distributions of ST incidence, including changes in seasonality and sex differences of ST incidence using nationally representative data. Unlike general perception [4–6], scrub typhus in South Korea exhibited a dual seasonal pattern, with larger peaks in autumn than in spring. Although the binomial pattern could derive from distribution of different species of mites by regions [18], as seen in Japan [19], it is possible that the dynamics of rodent populations might partially explain this bimodal but autumn-dominant pattern. The study conducted at Yongsan Garrison, Seoul, South Korea, from 2001 to 2005 indicated that the peak season for rodent populations was late summer, followed by autumn and early winter [20], when dominant ST seasonality was present. Rodent density had a temporally positive association with human ST cases in Guangzhou, China, in 2006–2014, which also had a bimodal ST pattern [21]. ST patients were more likely to observe rodents at home or at work compared to a control group in Darjeeling, India [22]. Furthermore, in South Korea, transmission of leptospirosis and hemorrhagic fever with renal syndrome, both unrelated to mites, shares similarities with ST in having an autumn peak and rodents as the main host [23, 24]. However, despite the requirement of a One Health framework for investigating human ST cases, studies in South Korea still have limitations in directly linking rodent populations to human ST cases. Therefore, continued surveillance of rodent distribution in relation to human ST cases is crucial to fully understand this causal relationship.

The changes in the strength and periodicity of seasonality suggest a potential impact of climate change. Comparing data from around 2013, we observed that the strength of the spring peak (26-week seasonality) increased regardless of sex or age. In South Korea, the chigger index in the summer increased from 2006 to 2007 [25]. Vector-borne diseases are expected to be modified because of climate change [26]. Although various factors, including urbanization, can result in seasonal changes in ST [5, 27, 28], climate change, which is affected by global warming, is postulated to alter the dynamics of diseases, including host behavior and timing of reproduction, by affecting complex interactions among reservoirs (rodents), vectors (mites), and incidental hosts (humans), ultimately modifying ST epidemiological patterns [7, 8]. Specifically, alterations to warming climate have extended and intensified the summer season in South Korea, consequently enhancing the survival and reproductive rates of ticks and rodents and increased engagement in outdoor activities of human [29]. These dynamics could lead to a heightened risk of tick and rodent exposure human population [29]. Previous studies have suggested a possible association between increased ST incidence and meteorological factors, which could indicate the effects of climate change [21, 30, 31]. Additionally, climate change factors beyond temperature, such as changes in humidity and precipitation patterns, could also impact vector and rodent populations by creating more favorable conditions for their survival and increasing the likelihood of disease transmission [32]. A study conducted in mainland China from 2012 to 2020 also proposed that changes in local climatic factors resulting from global climate dynamics could affect the environment, which is related to rodent survival [33]. Similarly, research indicates that tick-borne infections could also be associated with the impacts of climate shifts, as changes in climate influence the geographic distribution, population density, and seasonal activity of tick vectors, ultimately affecting disease transmission dynamics [34].

Moreover, the possibility of year-round ST prevalence in South Korea because of climate change cannot be ruled out. The observed reduction in the lengths of the 26- and 52-week periodicities suggests a potential shift toward a year-round prevalence. Climate change could directly impact life cycle of vector in accelerating development rates, meaning that different life stages may become active simultaneously [29]. The overlap in activity of nymphs and larvae increase the likelihood of pathogen transmission, as infected nymphs pass pathogens to larvae through a process known as non-systemic cofeeding, where the pathogen is transmitted directly between vectors feeding closely together on the same natural host—typically small mammals [29]. Previous research reveals increasing uncertainty in the seasonal peaks for adult ticks, nymphs, and larvae post-2019 [35]. Some tropical mite species exhibit multiple generations within a single year, whereas a single generation is common in temperate zones [36]. Therefore, continuous monitoring is necessary to plan appropriate interventions in response to climate change, which is caused by global warming [37].

Although the disease is still more prevalent in females, sex differences in ST incidence are decreasing in South Korea. The male-to-female ratio significantly increased, and the extent of autumn seasonality decreased in females and increased in males. This suggests that ST in South Korea is becoming aligned with the non-sex-specific nature of the disease, although the exact reasons for this change remain unknown. Notably, the decrease in autumn seasonality and increase in spring seasonality among females could be partially attributed to improved health behaviors specific to autumn, including agricultural behaviors. Specifically, in South Korea, female workers adopt a squatting position while working in dry fields, whereas male farmers stand and use tools in rice fields [1, 3, 38].

Patients, their families, and high-risk groups in the community, including farmers and garden workers have received education on preventing tick- and rodent-borne infectious diseases [11]. However, a more specific target should be established, as there was a different sex pattern by age group, particularly in the 40–59 and 60–79 age groups, with higher ST incidence among females and the male-dominant 20–39 age group. Further research is needed to understand the reasons for South Korea-specific sex differences and their temporal changes.

Our findings quantitatively demonstrated an increase in the power of spring seasonality, along with a decrease in overall periodicity and sex differences in ST in South Korea. Our study has several limitations that should be considered. Firstly, while we utilized comprehensive national health insurance data, these records primarily serve for billing purposes rather than solely for epidemiological research [9, 10]. Thus, variations in diagnostic practices, such as heightened physician awareness, or continuous campaigns to prevent ST infections may have influenced our findings [10]. Despite this limitation, the consistency in disease classification codes and health insurance billing criteria, along with the representativeness of our dataset with sex stratification, provided valuable insights into the long-term trends of ST in South Korea. Secondly, the ecological nature of our study design restricts causal inference. Although we identified significant temporal patterns and associations, the observed relationships do not imply causation. Lastly, our analysis did not incorporate detailed spatial data. Understanding the spatial distribution of scrub typhus cases and considering environmental factors could enhance our understanding within a One Health framework [36]. Future studies should aim to integrate spatio-temporal analyses and environmental data to elucidate the complex interactions among reservoirs, vectors, and human hosts. Despite these limitations, our study contributes valuable insights into the epidemiological trends of ST in South Korea, highlighting the importance of continued surveillance of rodent populations and targeted interventions to mitigate the impact of this vector-borne disease, especially in the context of changing climatic conditions [8].

## Electronic supplementary material

Below is the link to the electronic supplementary material.

Supplementary Material 1

Supplementary Material 2

Supplementary Material 3

Supplementary Material 4

Supplementary Material 5

Supplementary Material 6

## Data Availability

The data that support the findings of this study are available from the Korean National Health Insurance Service but restrictions apply to the availability of these data, which were used under license for the current study, and so are not publicly available. Data are however available from the authors upon reasonable request and with permission of the Korean National Health Insurance Service.
