# Supplementary material for: Changes in seasonality and sex ratio of scrub typhus: a case study of South Korea from 2003 to 2019 based on wavelet transform analysis
Source: BMC Infect Dis. 2024 Sep 28;24:1066. doi: 10.1186/s12879-024-09858-0 (PMC11438051; doi:10.1186/s12879-024-09858-0)
Supplement: Supplementary file 1 — Supplementary Material 1 [file 12879_2024_9858_MOESM1_ESM.docx]

**Table S1** Scrub typhus incidence per 100,000 population by age group

|  | Case count, N (%) | | | | | Incidence per 100000 population | | | | | |
| --- | --- | --- | --- | --- | --- | --- | --- | --- | --- | --- | --- |
| Year | 0–19 years of age | 20–39 years of age | 40–59 years of age | 60–79 years of age | ≥80 years of age | 0–19 years of age | 20–39 years of age | 40–59 years of age | 60–79 years of age | ≥80 years of age |  |
| 2003 | 179 (4.1) | 557 (12.7) | 1464 (33.3) | 2039 (46.4) | 157 (3.6) | 1.4 | 3.3 | 11.5 | 38.5 | 26.2 |  |
| 2004 | 369 (3.3) | 1234 (11.2) | 4019 (36.4) | 4980 (45.1) | 432 (3.9) | 2.9 | 7.4 | 30.6 | 91.1 | 69.1 |  |
| 2005 | 312 (2.6) | 1184 (10.0) | 3989 (33.8) | 5694 (48.3) | 618 (5.2) | 2.5 | 7.2 | 29.3 | 101.6 | 93.7 |  |
| 2006 | 283 (2.8) | 974 (9.5) | 3455 (33.8) | 4947 (48.3) | 578 (5.6) | 2.3 | 6.0 | 24.6 | 85.2 | 83.0 |  |
| 2007 | 201 (2.5) | 691 (8.5) | 2713 (33.4) | 3933 (48.5) | 575 (7.1) | 1.7 | 4.3 | 18.9 | 64.9 | 77.3 |  |
| 2008 | 299 (2.6) | 944 (8.3) | 3797 (33.2) | 5624 (49.2) | 760 (6.7) | 2.5 | 6.0 | 25.6 | 89.3 | 95.3 |  |
| 2009 | 422 (2.7) | 1356 (8.7) | 5293 (34.1) | 7385 (47.6) | 1046 (6.7) | 3.6 | 8.8 | 34.7 | 113.2 | 121.6 |  |
| 2010 | 351 (2.7) | 1125 (8.5) | 4570 (34.5) | 6305 (47.7) | 879 (6.6) | 3.0 | 7.4 | 28.7 | 92.6 | 93.3 |  |
| 2011 | 369 (2.7) | 1108 (8.1) | 4703 (34.3) | 6516 (47.6) | 1004 (7.3) | 3.2 | 7.4 | 28.7 | 93.2 | 97.0 |  |
| 2012 | 514 (2.7) | 1480 (7.8) | 6452 (34.1) | 9076 (47.9) | 1420 (7.5) | 4.6 | 10.0 | 38.8 | 124.3 | 127.6 |  |
| 2013 | 374 (2.0) | 1279 (6.8) | 6435 (34.2) | 9205 (48.9) | 1529 (8.1) | 3.4 | 8.8 | 38.0 | 122.0 | 127.9 |  |
| 2014 | 270 (1.9) | 1005 (7.1) | 4423 (31.5) | 7020 (49.9) | 1345 (9.6) | 2.6 | 7.0 | 25.8 | 89.4 | 103.9 |  |
| 2015 | 280 (1.8) | 1055 (6.7) | 4671 (29.7) | 8038 (51.1) | 1675 (10.7) | 2.7 | 7.3 | 27.2 | 97.4 | 119.0 |  |
| 2016 | 338 (2.0) | 1173 (6.9) | 5176 (30.3) | 8637 (50.6) | 1762 (10.3) | 3.4 | 8.2 | 30.1 | 100.2 | 116.0 |  |
| 2017 | 265 (2.0) | 952 (7.2) | 3864 (29.1) | 6692 (50.5) | 1489 (11.2) | 2.7 | 6.7 | 22.5 | 74.1 | 91.4 |  |
| 2018 | 166 (1.9) | 617 (6.9) | 2389 (26.9) | 4535 (51.1) | 1172 (13.2) | 1.8 | 4.4 | 14.0 | 48.0 | 67.1 |  |
| 2019 | 142 (1.8) | 522 (6.7) | 1972 (25.4) | 4002 (51.5) | 1132 (14.6) | 1.6 | 3.8 | 11.6 | 40.4 | 60.0 |  |
| Total | 5134 (2.4) | 17256 (8.1) | 69385 (32.4) | 104628 (48.9) | 17573 (8.2) | 2.7 | 6.7 | 26.0 | 85.2 | 93.7 |  |
