# Supplementary material for: Changes in seasonality and sex ratio of scrub typhus: a case study of South Korea from 2003 to 2019 based on wavelet transform analysis
Source: BMC Infect Dis. 2024 Sep 28;24:1066. doi: 10.1186/s12879-024-09858-0 (PMC11438051; doi:10.1186/s12879-024-09858-0)
Supplement: Supplementary file 2 — Supplementary Material 2 [file 12879_2024_9858_MOESM2_ESM.docx]

|  | 0–19 years of age | | 20–39 years of age | | 40–59 years of age | | 60–79 years of age | | ≥80 years of age | | Male-to-female ratio of incidence per 100,000 population by age group  (years of age) | | | | |
| --- | --- | --- | --- | --- | --- | --- | --- | --- | --- | --- | --- | --- | --- | --- | --- |
| Year | Male | Female | Male | Female | Male | Female | Male | Female | Male | Female | 0–19 | 20–39 | 40–59 | 60–79 | ≥80 |
| 2003 | 92 (51.4) | 87 (48.6) | 287 (51.5) | 270 (48.5) | 594 (40.6) | 870 (59.4) | 752 (36.9) | 1287 (63.1) | 51 (32.5) | 106 (67.5) | 1.0 | 1.0 | 0.7 | 0.8 | 1.0 |
| 2004 | 206 (55.8) | 163 (44.2) | 679 (55.0) | 555 (45.0) | 1562 (38.9) | 2457 (61.1) | 1743 (35.0) | 3237 (65.0) | 165 (38.2) | 267 (61.8) | 1.1 | 1.2 | 0.6 | 0.7 | 1.3 |
| 2005 | 171 (54.8) | 141 (45.2) | 628 (53.0) | 556 (47.0) | 1510 (37.9) | 2479 (62.1) | 1939 (34.1) | 3755 (65.9) | 209 (33.8) | 409 (66.2) | 1.1 | 1.1 | 0.6 | 0.7 | 1.1 |
| 2006 | 143 (50.5) | 140 (49.5) | 508 (52.2) | 466 (47.8) | 1315 (38.1) | 2140 (61.9) | 1793 (36.2) | 3154 (63.8) | 185 (32.0) | 393 (68.0) | 0.9 | 1.0 | 0.6 | 0.7 | 1.0 |
| 2007 | 107 (53.2) | 94 (46.8) | 378 (54.7) | 313 (45.3) | 1076 (39.7) | 1637 (60.3) | 1382 (35.1) | 2551 (64.9) | 171 (29.7) | 404 (70.3) | 1.0 | 1.2 | 0.6 | 0.7 | 0.9 |
| 2008 | 165 (55.2) | 134 (44.8) | 494 (52.3) | 450 (47.7) | 1520 (40.0) | 2277 (60.0) | 2009 (35.7) | 3615 (64.3) | 232 (30.5) | 528 (69.5) | 1.1 | 1.0 | 0.7 | 0.7 | 1.0 |
| 2009 | 221 (52.4) | 201 (47.6) | 759 (56.0) | 597 (44.0) | 2180 (41.2) | 3113 (58.8) | 2664 (36.1) | 4721 (63.9) | 310 (29.6) | 736 (70.4) | 1.0 | 1.2 | 0.7 | 0.7 | 0.9 |
| 2010 | 200 (57.0) | 151 (43.0) | 617 (54.8) | 508 (45.2) | 1918 (42.0) | 2652 (58.0) | 2390 (37.9) | 3915 (62.1) | 240 (27.3) | 639 (72.7) | 1.2 | 1.1 | 0.7 | 0.7 | 0.9 |
| 2011 | 214 (58.0) | 155 (42.0) | 617 (55.7) | 491 (44.3) | 1975 (42.0) | 2728 (58.0) | 2374 (36.4) | 4142 (63.6) | 285 (28.4) | 719 (71.6) | 1.3 | 1.2 | 0.7 | 0.7 | 1.0 |
| 2012 | 293 (57.0) | 221 (43.0) | 808 (54.6) | 672 (45.4) | 2804 (43.5) | 3648 (56.5) | 3426 (37.7) | 5650 (62.3) | 435 (30.6) | 985 (69.4) | 1.2 | 1.1 | 0.7 | 0.7 | 1.1 |
| 2013 | 218 (58.3) | 156 (41.7) | 734 (57.4) | 545 (42.6) | 2720 (42.3) | 3715 (57.7) | 3610 (39.2) | 5595 (60.8) | 460 (30.1) | 1069 (69.9) | 1.3 | 1.3 | 0.7 | 0.8 | 1.0 |
| 2014 | 149 (55.2) | 121 (44.8) | 590 (58.7) | 415 (41.3) | 1921 (43.4) | 2502 (56.6) | 2633 (37.5) | 4387 (62.5) | 412 (30.6) | 933 (69.4) | 1.1 | 1.3 | 0.7 | 0.7 | 1.0 |
| 2015 | 146 (52.1) | 134 (47.9) | 600 (56.9) | 455 (43.1) | 1980 (42.4) | 2691 (57.6) | 3042 (37.8) | 4996 (62.2) | 518 (30.9) | 1157 (69.1) | 1.0 | 1.2 | 0.7 | 0.7 | 1.0 |
| 2016 | 190 (56.2) | 148 (43.8) | 688 (58.7) | 485 (41.3) | 2377 (45.9) | 2799 (54.1) | 3400 (39.4) | 5237 (60.6) | 536 (30.4) | 1226 (69.6) | 1.2 | 1.3 | 0.8 | 0.7 | 1.0 |
| 2017 | 153 (57.7) | 112 (42.3) | 544 (57.1) | 408 (42.9) | 1769 (45.8) | 2095 (54.2) | 2680 (40.0) | 4012 (60.0) | 462 (31.0) | 1027 (69.0) | 1.3 | 1.2 | 0.8 | 0.8 | 1.0 |
| 2018 | 86 (51.8) | 80 (48.2) | 377 (61.1) | 240 (38.9) | 1134 (47.5) | 1255 (52.5) | 1822 (40.2) | 2713 (59.8) | 350 (29.9) | 822 (70.1) | 1.0 | 1.5 | 0.9 | 0.8 | 0.9 |
| 2019 | 76 (53.5) | 66 (46.5) | 328 (62.8) | 194 (37.2) | 982 (49.8) | 990 (50.2) | 1650 (41.2) | 2352 (58.8) | 351 (31.0) | 781 (69.0) | 1.1 | 1.6 | 1.0 | 0.8 | 0.9 |
| Total | 2830 (55.1) | 2304 (44.9) | 9636 (55.8) | 7620 (44.2) | 29337 (42.3) | 40048 (57.7) | 39309 (37.6) | 65319 (62.4) | 5372 (30.6) | 12201 (69.4) | 1.1 | 1.2 | 0.7 | 0.7 | 1.0 |

**Table S2** Scrub typhus case count and male-to-female ratio of incidence per 100,000 population by year and age group
