# Supplementary figures and images for: Changes in seasonality and sex ratio of scrub typhus: a case study of South Korea from 2003 to 2019 based on wavelet transform analysis
Source: BMC Infect Dis. 2024 Sep 28;24:1066. doi: 10.1186/s12879-024-09858-0 (PMC11438051; doi:10.1186/s12879-024-09858-0)

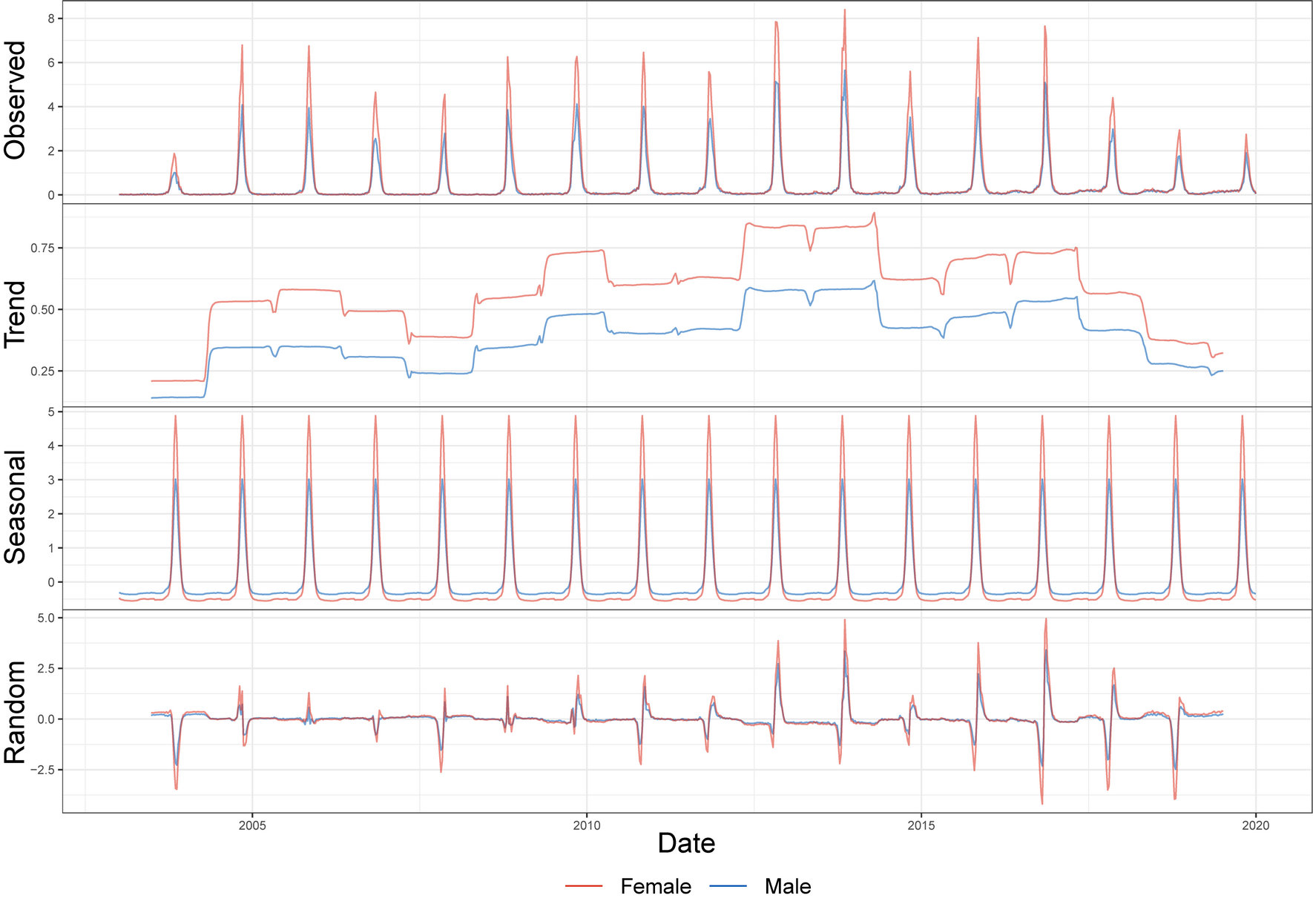

Supplement: Supplementary file 3 — Supplementary Material 3 [file 12879_2024_9858_MOESM3_ESM.tif]

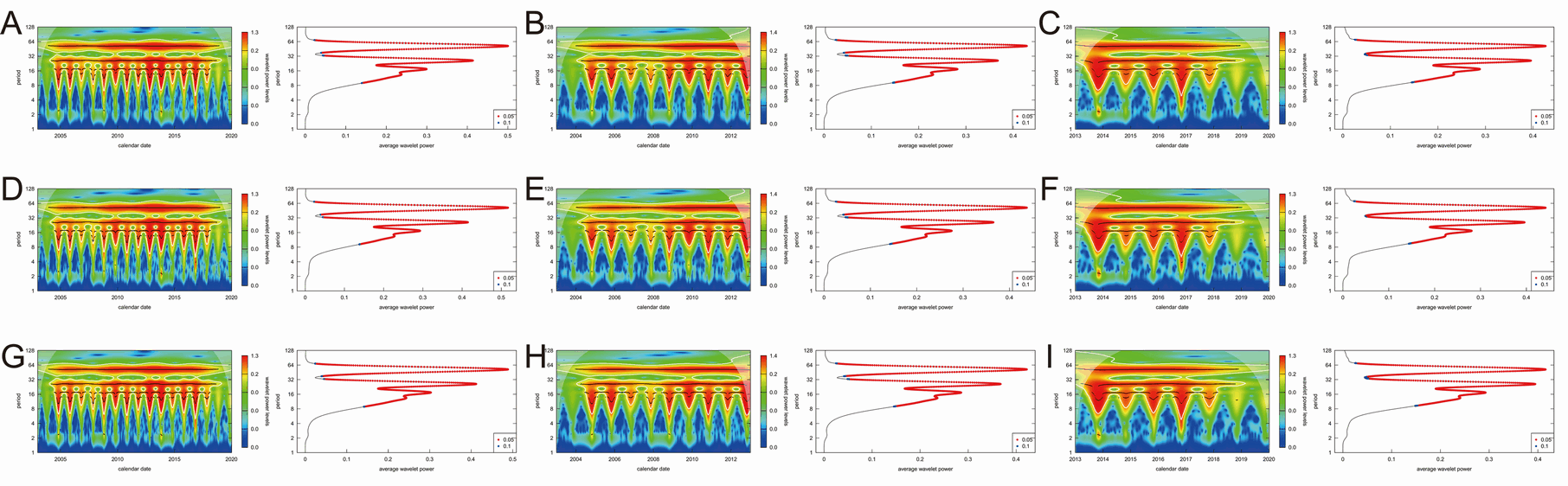

Supplement: Supplementary file 4 — Supplementary Material 4 [file 12879_2024_9858_MOESM4_ESM.tif]

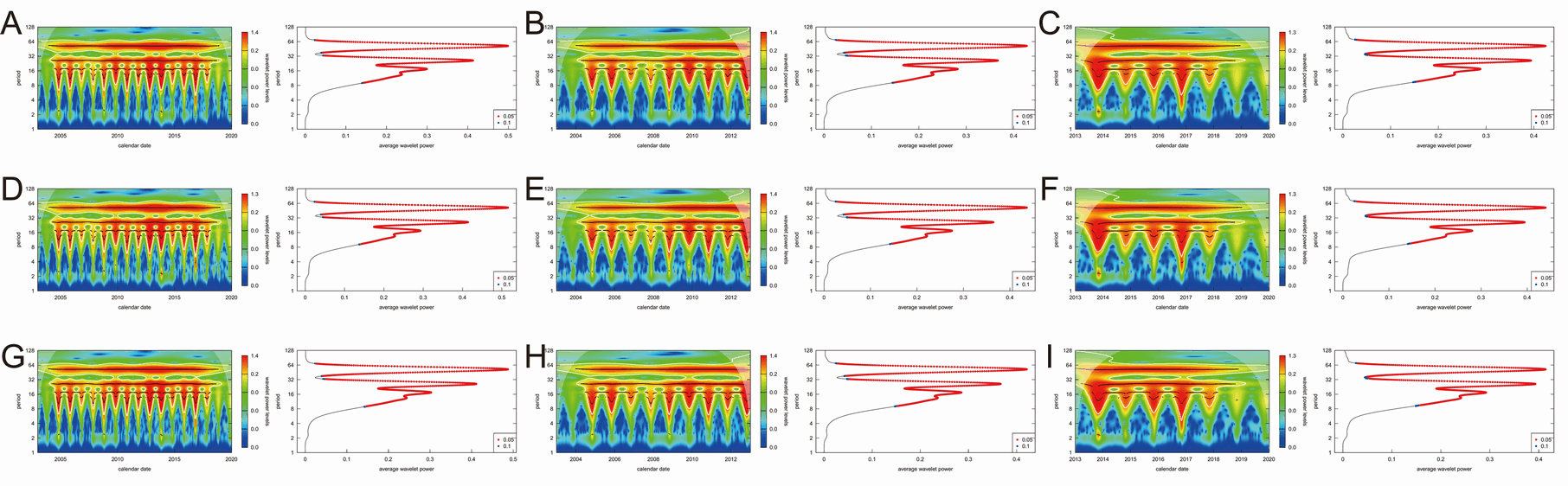

Supplement: Supplementary file 5 — Supplementary Material 5 [file 12879_2024_9858_MOESM5_ESM.tif]

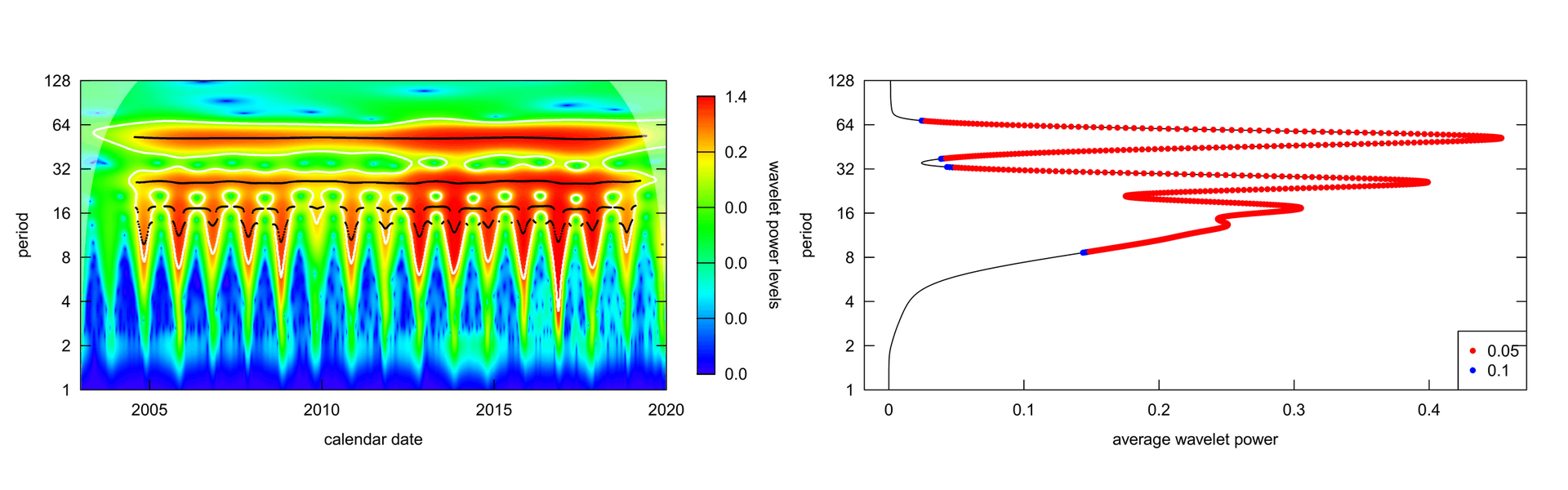

Supplement: Supplementary file 6 — Supplementary Material 6 [file 12879_2024_9858_MOESM6_ESM.tif]
